# How relationship status and sociosexual orientation influence the link between facial attractiveness and visual attention

**DOI:** 10.1371/journal.pone.0207477

**Published:** 2018-11-14

**Authors:** Aleksandra Mitrovic, Juergen Goller, Pablo P. L. Tinio, Helmut Leder

**Affiliations:** 1 Department of Basic Psychological Research and Research Methods, University of Vienna, Vienna, Austria; 2 College of Education and Human Services, Montclair State University, Montclair, New Jersey, United States of America; Universita degli Studi di Verona, ITALY

## Abstract

Facial attractiveness captures and binds visual attention, thus affecting visual exploration of our environment. It is often argued that this effect on attention has evolutionary functions related to mating. Although plausible, such perspectives have been challenged by recent behavioral and eye-tracking studies, which have shown that the effect on attention is moderated by various sex- and goal-related variables such as sexual orientation. In the present study, we examined how relationship status and sociosexual orientation moderate the link between attractiveness and visual attention. We hypothesized that attractiveness leads to longer looks and that being single as well as being more sociosexually unrestricted, enhances the effect of attractiveness. Using an eye-tracking free-viewing paradigm, we tested 150 heterosexual men and women looking at images of urban real-world scenes depicting two people differing in facial attractiveness. Participants additionally provided attractiveness ratings of all stimuli. We analyzed the correlations between how long faces were looked at and participants’ ratings of attractiveness and found that more attractive faces—especially of the other sex—were looked at longer. We also found that more sociosexually unrestricted participants who were single had the highest attractiveness-attention correlation. Our results show that evolutionary predictions cannot fully explain the attractiveness-attention correlation; perceiver characteristics and motives moderate this relationship.

## Introduction

Attractiveness captures and binds visual attention and has a strong influence on the way we visually explore our environment [1–5]. This effect is especially strong in faces, which have outstandingly high biological and social relevance [6–9]. The finding that attractive faces are looked at longer than less attractive faces was shown in a number of eye-tracking experiments in the laboratory [10–15] and in experiments in real world settings, in which participants interacted with an attractive confederate [16–18]. While this effect has been shown to be moderated by sexual orientation [12, 19] and threat [11], the extent to which other socially relevant and personality variables moderate the effect is less clear. In this study, we examined how relationship status and sociosexual orientation and their interaction moderate the link between facial attractiveness and visual attention.

How can the effects of facial attractiveness on visual attention be explained? The most dominant explanation of the link between attractiveness and visual attention is related to mate selection [20, 21]. From this perspective, our attention is biased towards attractive people in order to facilitate sexual reproduction with those who are likely to contribute good genes to offspring. Therefore, one of the functions of the aesthetic sense is to detect indicators of good genes, with attractiveness being a primary indicator [22–26]. There is some evidence for this perspective. For example, facial attractiveness is negatively correlated with fluctuating asymmetry [27], which is an indicator of developmental instability. Preference for people with low fluctuating asymmetry is beneficial as such people presumably have good genes. In this way, facial attractiveness indicates developmental stability, and in turn, high likelihood of reproductive success. The issue with such explanation is that it only applies to other-sex faces.

One study testing such a preference for other-sex faces was conducted by Maner and colleagues [14]. They presented participants two sets (one for each sex) of eight isolated, same-sex faces of which four were selected to be attractive and four to be average-looking and recorded the extent to which participants selectively attended to attractive faces. They had three hypotheses. The “*opposite-sexed beauty captures the mind*” hypothesis, which states that “both men and women would selectively focus on (and, in turn, remember) highly attractive members of the opposite sex” [14] (p. 1108). Their *“female beauty captures the mind*” hypothesis states that “both men and women might exhibit processing advantages for attractive female targets—men because of mate-search motives, and women because of mate guarding or self-assessment motives" [14] (p. 1109). Their “*one-sided gender bias*” hypothesis states that “males, more than females, will selectively focus on (and, in turn, remember) attractive members of the opposite sex” [14] (p. 1109). They found that visual attention was highest for female faces, which is support for the “*female beauty captures the mind”* hypothesis and in their eye-tracking study found additional support for the “*opposite-sexed beauty captures the mind”* hypothesis.

In our study, we expected to replicate these findings. However, these effects only partly explain how visual attention works in everyday life, and evolutionary perspectives cannot easily account for visual attention towards faces. If indeed mating for reproduction underlies visual attention to faces, then only highly attractive, other-sex people would receive attention. However, this is not the case as less attractive people also receive, and oftentimes, bind the perceivers’ attention. Thus, it is very unlikely that reproduction is the only influencing force behind the link between attractiveness and visual attention.

Many person-level characteristics have been shown to moderate the attractiveness-attention correlation. For example, attentional biases towards attractive male faces diminish when the perceiver is put in a threatening state of mind [11]—an effect that can be explained by our evolutionary past. Although it was generally important to identify highly attractive partners, in a threatening life or death situation—as when being chased by a predator—the drive to survive overrides the mating-driven function of visual attention. A further—probably stronger—evidence against the evolutionary perspective is research that has taken into account the sexual orientation of perceivers. Eye-tracking studies that have compared the visual attention of homosexual and heterosexual perceivers showed that perceivers looked longer at people of their preferred sex [12, 28]. This finding directly challenges the strict evolutionary notion that people are primarily motivated by a drive to reproduce and consequently should visually prefer attractive faces of the *other* sex.

Another motivation-related factor that likely moderates the attractiveness-attention correlation is relationship status. If the primary function of facial attractiveness is to facilitate the spread of genes, it should make no difference whether a perceiver of highly attractive faces already has a partner to reproduce with. Evolutionary notions would predict that people would still look for more and better opportunities to reproduce. Otherwise, if the attention binding effects of attractiveness are driven by a need to find a partner, and not necessarily the spread of genes, other attractive faces should be of less importance for people who already are committed to a partner. In committed people, the goal to find a partner (with whom to reproduce) should essentially be fulfilled. After all, the “need” for a partner has been satisfied and the relationship goal has been met [10]. The evolutionary perspective could be critically tested if people who are in a romantic relationship and people who are single show the same attractiveness-attention correlation. Thus, we hypothesized shorter looking durations for attractive other-sex faces by people in a romantic relationship.

The finding that attractive other-sex faces are looked at more briefly by people who are in a romantic relationship would be in accordance with the *relationship maintenance* phenomenon [29–31] according to which people perceive attractive other-sex faces as potentially threatening alternatives to their current relationship partner. Then, being exposed to attractive, other-sex faces could lead to a decrease in: (a) satisfaction with one’s relationship, (b) relationship commitment, and (c) the perceived attractiveness of the current partner [32–35]. Consequently, people who are already in a relationship might devalue the attractiveness of alternatives in order to not endanger their satisfaction with their current partners [30, 36, 37–44]. Following this argument, we expected that the effects of attractiveness on attention would be generally weaker for people who are in a romantic relationship and would clearly be stronger for people who are single.

Another person-level characteristic that is relevant to mating is sociosexual orientation. Sociosexual orientation is a personality construct that indicates the degree of emotional commitment needed before a person engages in sexual relationships [45, 46]. More sociosexually unrestricted people need less emotional commitment to engage in sexual relationships. Men often show higher scores on questionnaires regarding sociosexual orientation, which means that they are, on average, more sociosexually unrestricted than women [47–51]. In contrast to less sociosexually unrestricted people who tend to have long-term relationships, more sociosexually unrestricted people are prone to having many short-term relationships [52]. Attractiveness is more important for men [53–56] and for short-term relationships [57–60], both correlating with a more unrestricted sociosexual orientation. Therefore, regarding mate selection, one might assume that for more sociosexually unrestricted people who tend to have more short-term relationships and who are more likely male, attractiveness in a partner is more important than for less sociosexually unrestricted people [61]. Indeed, more sociosexually unrestricted people, especially men, allocate more of their attention to attractive other-sex faces [14, 40, 62, 63]. Thus, we hypothesized that the attractiveness-attention correlation would be stronger for more sociosexually unrestricted than less sociosexually unrestricted people.

To our knowledge, the only eye-tracking study that has included sex, relationship status, and sociosexual orientation was conducted by Maner and colleagues [14]. They found evidence for the “*opposite-sexed beauty captures the mind*” hypothesis and the “*female beauty captures the mind*” hypothesis. They also found that more sociosexually unrestricted people looked longer at faces of the other sex and that relationship status only had an influence on women. However, in their study, sex of the perceiver, sex of the face, relationship status, and sociosexual orientation were not analyzed in one statistical model. Therefore, the interactions of these factors are unknown. In the present study, we analyzed the individual effects of these factors as well as their interactions in a full-factorial design. We were especially interested in the interaction of relationship status and sociosexual orientation. If they interact, the attractiveness-attention correlation would be the strongest for single and more sociosexually unrestricted participants. Otherwise, we expected that the attractiveness-attention correlation would be stronger for single and for more sociosexually unrestricted people.

To sum up our hypotheses, we expected participants to generally look longer at more attractive than less attractive faces [10, 11, 12–14]. In line with the “*opposite-sexed beauty captures the mind*” hypothesis [14], we expected an interaction between sex of the face stimulus and sex of the perceiver. Consistent with the literature [14, 53, 55, 56, 64, 65], we expected this effect to be most pronounced for men looking at female faces (“*one-sided gender bias*”). We expected higher correlations between attractiveness and attention for single participants and those who are more sociosexually unrestricted [10, 14, 40, 62]. If there is an interaction between sociosexual orientation and relationship status, then we would expect that more sociosexually unrestricted, single participants would spend more time looking at attractive faces. According to the strict evolutionary line of argument, these moderating effects should apply to other-sex faces and should be stronger for men than for women.

In the present study, we examined how the effects of facial attractiveness on visual attention are moderated by the sex of the face stimulus and the perceiver’s sex, relationship status, and sociosexual orientation. Each participant rated each face stimulus for attractiveness on a Likert scale. We combined these explicit ratings with measures of eye movements, which reflect more automatic and implicit behavioral responses to facial attractiveness. We employed a free-viewing paradigm in which the participant viewed the faces without having to perform a task. This paradigm provides an unobtrusive way to examine visual attention towards faces [10, 14, 28].

We used images of urban real-world street scenes showing two people instead of presenting individual faces or arrays of faces [14]. Beyond pairs of same-sex faces (female-female and male-male), we also included mixed-sex scenes (male-female). Because we tested heterosexual participants, the different sets had different implications for our hypotheses. The scenes with different sex faces than the perceiver’s sex corresponded to the hypothesis of mate selection. However, mixed-sex scenes are more suitable for directly testing the idea of devaluing potential competitors of the same sex. Moreover, most empirical studies on facial attractiveness compared highly attractive faces with less/non-attractive faces based on pre-ratings [1, 3]. This pre-categorization of faces into attractive versus non-attractive faces does not allow the testing of individual variation in taste among participants [66, 67] and does not reflect natural variation in attractiveness. Therefore, we used the individual attractiveness ratings for our analyses to predict individual eye movements. To minimize possible transfer effects between ratings of male and female faces [68], we employed a block-by-block presentation according to scene type (male-male, female-female, male-female).

Taken together, we studied the moderating effects of relationship status and sociosexual orientation on the attractiveness-attention correlation and expected that the relationship would be stronger for single as compared to people in a relationship and for more sociosexually unrestricted as compared to less sociosexually unrestricted people. Moreover, we expected that the relationship would be strongest for single people who are more sociosexually unrestricted.

## Method

### Participants

One hundred ninety one undergraduate students participated for course credit. We excluded 22 participants because they indicated a non-heterosexual sexual orientation (10 bisexual women, 4 bisexual men, 2 homosexual women, 5 homosexual men, 1 other) and 19 participants because of problems during data collection. The final sample consisted of 150 participants (77 women, 73 men, *M*_age_ = 22.45 years, *SD*_age_ = 3.06, age range: 18–36 years). In a post-questionnaire, 81 participants (54%: 45 women, 36 men) indicated that they were in a relationship at the time of the study and 69 (46%: 32 women, 37 men) indicated that they were single. The average score for sociosexual orientation was *M* = 5.11 for men (*SD* = 1.47, *Mdn* = 5.22, range: 1.89–8.67) and *M* = 4.03 for women (*SD* = 1.38, *Mdn* = 3.67, range: 1.67–7.56), which was measured on a 9-point Likert scale, with higher ratings indicating more unrestricted sociosexual orientation. These values are in line with previous findings [46]. All participants had normal or corrected-to-normal visual acuity and normal color vision. All participants provided written informed consent before they participated in the study, and the study was approved by the Ethics Committee of the University of Vienna.

### Materials

**Fig 1:**
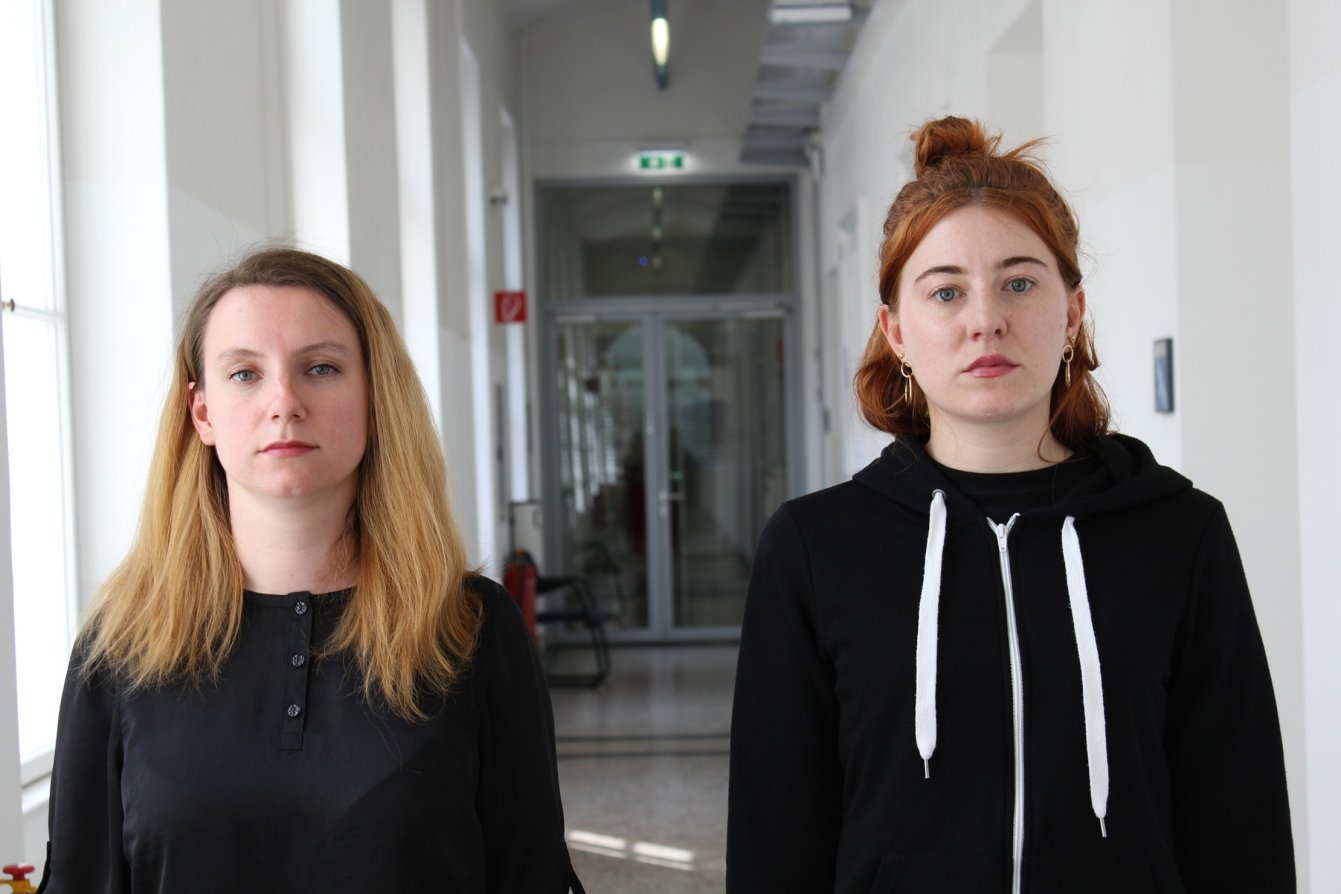
Stimulus example. Reenacted example of a target scene (not used in the actual study). We obtained written informed consent for publication from the individuals included in the figure.

We used 45 images of urban, real-world street scenes showing two college-aged women (15 scenes), men (15 scenes), or one woman and one man (15 scenes) standing next to each other. We instructed models to switch positions (left-right); therefore, two photos were taken of each of the pairs. Each model was randomly allocated to one scene resulting in a range of combinations regarding facial attractiveness. The models looked directly into the camera and were instructed to show a neutral facial expression (see Fig 1 for a sample image; the depicted individuals gave written informed consent to publish their pictures as outlined in the PLOS consent form). For each face, we defined a circular area of interest covering the whole face (the area used for analyzing the eye movement data). Because the size of the heads varied from face to face between scenes, the diameter of the areas of interest ranged from 180 pixels (4° visual angle) to 250 pixels (6°). However, the sizes were the same for both faces within each scene. To further conceal the aim of the study, we presented 50 distractor images. The distractor images showed similar backgrounds as the actual test scenes, but depicted only one person or two objects replacing the two people (e.g., two posters hanging next to each other on a wall). All images were shown in full color and scaled to 1200 pixels (28°) × 800 pixels (19°).

Eye movement data were collected using an EyeLink 1000 desktop-mounted eye tracker (SR Research Ltd., Mississauga, Ontario, Canada), sampling at 1000 Hz. Viewing was binocular but only the dominant eye was tracked. The scenes were shown on a 24-inch LCD-monitor (Samsung SyncMaster 2443BW, 16:9, 1920 × 1200, 60 Hz) and viewed from a distance of 65 cm. To facilitate accurate measurements and minimize movements, we used a chin rest to stabilize participants’ head positions. We used a 9-point calibration and validation of the eye tracker. The study was implemented using Experiment Builder software (Version 1.10.1630, SR Research Ltd., Mississauga, Ontario, Canada). Data analyses were based on the fixation report provided by the DataViewer software (Version 2.3.22, SR Research Ltd., Mississauga, Ontario, Canada).

### Procedure and design

The study consisted of three blocks: an eye-tracking block and two subsequent behavioral rating blocks. In the eye-tracking block, all 95 images (45 scenes + 50 distractor images) were presented in random order for 10 seconds each. Between the images, a fixation cross was presented at the center of the screen and on which participants were required to fixate to trigger the presentation of the next image. This fixation check was used to assess the quality of the eye-tracking signal. If there was a drift in the signal, recalibration and validation were performed. Participants were instructed to freely view the scenes without having a specific task.

In the second block, the 45 scenes were presented again, but this time blocked in terms of female, male, and mixed-sex scenes. Participants rated each face for attractiveness on a 7-point Likert scale ranging from 1 (*not attractive*) to 7 (*very attractive*). For each scene, both faces were rated, with the order regarding which of the two faces was rated first randomly changing from trial to trial. In the third block, all scenes were presented again and participants rated each face for whether they knew the face before the study. We deleted all trials in which participants indicated knowing at least one face in order to eliminate any confounding effects of familiarity on visual attention [69] or on attractiveness [70].

Participants then filled out a questionnaire about their age, sex, sexual orientation, relationship status, and relationship satisfaction. We also administered the revised Sociosexual Orientation Inventory (SOI-R) [46] that consists of nine items answered on a 9-point Likert scale. We used the mean value of these items as a score indicating participants’ sociosexual orientation. A lower score indicated that the participant was less sociosexually unrestricted, whereas a higher score indicated that the participant was more sociosexually unrestricted. We administered the questionnaires at the end of the study to avoid making participants’ relationship status salient, as this could have influenced participants’ behavior. Finally, participants were debriefed about the aim of the study and thanked for their participation.

## Results

All data are freely available under http://phaidra.univie.ac.at/o:756919. As an indicator of facial attractiveness, we analyzed the individual ratings given in the second block. As an indicator of visual attention, we calculated the total fixation duration (TFD) defined as the summed dwell time for each face (area of interest) in milliseconds (*M*_TFD_ = 2893.54 ms, *SD*_TFD_ = 1247.66 ms). We did not analyze saccades or blinks and excluded all fixations that were shorter than 100 ms and all trials in which participants indicated knowing at least one of the two faces in the scene. We were interested in how the link between attractiveness and visual attention is moderated by other key variables. To operationalize this relationship, we calculated Pearson correlation coefficients between attractiveness and TFD for each participant separately for female and male face stimuli. Table 1 shows the correlation coefficients separately for same-sex and mixed-sex scenes.

**Table 1:** Mean correlation coefficients between TFD and attractiveness.

	Same-sex scenes			Mixed-sex scenes			Total		
Factors	Female	Male	Total	Female	Male	Total	Female	Male	Total
Total	.23	.08	.16	.19	.13	.16	.21	.11	.16
Men	.32	.05	.19	.23	.09	.16	.27	.07	.17
Committed	.33	.09	.21	.22	.08	.15	.27	.08	.18
Restricted	.30	.14	.22	.19	.01	.10	.24	.08	.16
Unrestricted	.35	.03	.19	.26	.15	.20	.31	.09	.20
Single	.32	.02	.17	.23	.10	.17	.27	.06	.17
Restricted	.25	.00	.12	.19	.17	.18	.22	.09	.15
Unrestricted	.38	.04	.21	.27	.03	.15	.32	.04	.18
Women	.14	.11	.13	.15	.17	.16	.15	.14	.14
Committed	.13	.11	.12	.18	.14	.16	.16	.13	.14
Restricted	.13	.12	.12	.11	.06	.08	.12	.09	.10
Unrestricted	.13	.11	.12	.27	.26	.26	.20	.18	.19
Single	.16	.11	.13	.11	.21	.16	.13	.16	.14
Restricted	.07	.07	.07	.08	.24	.16	.08	.16	.12
Unrestricted	.23	.13	.18	.13	.18	.15	.18	.16	.17

*Note*. Sociosexual orientation was calculated by a median split, separately for women (*Mdn* = 3.78) and men (*Mdn* = 5.22). Please note that a median split is not recommended by Penke and Asendorpf [46] to avoid categorizing people into “restricted” and “unrestricted”. However, that was the only way to illustrate the results in this table. For the actual analyses, we used the individual values and made no median split.

To test how relationship status and sociosexual orientation moderate the attractiveness-attention correlation, we ran two linear mixed models (LMM) with the correlation coefficients as dependent variable, separately for same-sex (see deposited data “o:756900”) and mixed-sex (see deposited data “o:756904”) scenes using the lme4 package (version 1.1–8) [71] in R (version 3.1.0, R Development Core Team; Satterthwaite approximations for *p*-values). We defined contrasts for the fixed effects of the sex of the participant (women–men), sex of face stimulus (female–male), and relationship status (single–committed). Sociosexual orientation was included as a centered, continuous fixed effect. Interactions among all factors were included. Random by-participant intercepts and slopes for sex of face and attractiveness were included. The results of the LMMs are shown in Table 2. We ran two additional LMMs (one per scene type) with TFD as the dependent variable. We explain these LMMs and their results in the supplementary materials section (S1 Appendix).

**Table 2:** Results for the LMM with correlation coefficients as dependent variable separately for same-sex and mixed-sex scenes.

	Same-sex scenes					Mixed-sex scenes				
	Est.	*SE*	*df*	*t*	*p*	Est.	*SE*	*df*	*t*	*p*
Intercept	0.16	0.02	122	9.75	< .001*	0.17	0.02	140	8.30	< .001*
Sex of the participant	-0.05	0.03	122	-1.69	.094†	0.02	0.04	140	0.58	.566
Sex of the face	0.14	0.03	122	4.83	< .001*	0.04	0.04	140	1.14	.255
Relationship status	-0.02	0.03	122	-0.75	.458	-0.01	0.04	140	-0.15	.883
Sociosexual orientation (SOI)	0.01	0.01	124	1.28	.202	0.02	0.01	140	1.33	.187
Sex of the participant : Sex of the face	-0.20	0.06	122	-3.51	< .001*	-0.17	0.07	140	-2.24	.027*
Sex of the participant : Relationship status	0.08	0.06	122	1.29	.201	-0.08	0.08	140	-0.91	.363
Sex of the face : Relationship status	0.05	0.06	122	0.84	.405	-0.08	0.07	140	-1.13	.259
Sex of the participant : SOI	0.01	0.02	124	0.59	.559	0.04	0.03	140	1.47	.143
Sex of the face : SOI	0.03	0.02	124	1.75	.083†	0.00	0.02	140	0.13	.898
Relationship status : SOI	0.05	0.02	124	2.15	.034*	-0.03	0.03	140	-1.18	.241
Sex of the participant : Sex of the face : Relationship status	-0.03	0.11	122	-0.23	.822	-0.06	0.15	140	-0.41	.680
Sex of the participant: Sex of the face : SOI	-0.05	0.04	124	-1.20	.231	-0.04	0.05	140	-0.77	.445
Sex of the participant : Relationship status : SOI	-0.01	0.04	124	-0.23	.822	0.00	0.06	140	-0.05	.961
Sex of the face : Relationship status : SOI	-0.00	0.04	124	-0.08	.933	0.05	0.05	140	1.06	.291
Sex of the participant : Sex of the face : Relationship status : SOI	0.02	0.08	124	0.24	.807	-0.06	0.10	140	-0.59	.559

Note.

* p < 0.05

† p < 0.10. SOI: Sociosexual orientation. Contrasts: sex of the participant (women–men); sex of the face (female faces–male faces); relationship status (single–committed).

### Same-sex scenes

Table 1 shows the correlation coefficients between the total fixation duration (TFD) and the attractiveness ratings for both sexes in both types of scenes as well as the scenes merged, for all combinations of sex, relationship status, and sociosexual orientation of the participants. Positive correlation coefficients indicate a positive link between attractiveness and visual attention such that an increase in attractiveness ratings is associated with longer fixation durations. It can be seen in Table 1 that all correlation coefficients for same-sex scenes were positive across all groups, indicating a general positive correlation between attractiveness and TFD.

**Fig 2:**
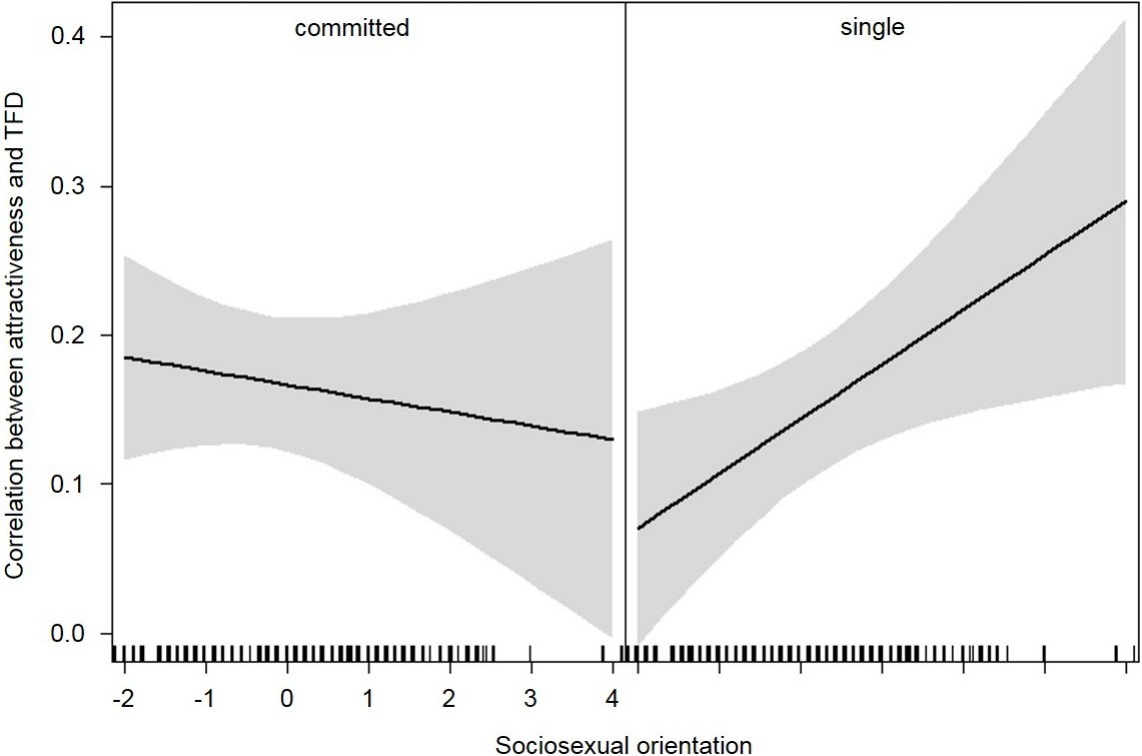
Relationship status × sociosexual orientation interaction (same-sex scenes). Relationship Status × Sociosexual Orientation interaction on the correlation between attractiveness and TFD in same-sex scenes.

We found an main effect of sex of face (*b* = 0.14, *SE* = 0.03, *t*(122) = 4.83, *p* < .001) with the correlation being generally stronger for female (*r* = .23) than for male faces (*r* = .08). We found a Participant Sex × Sex of Face interaction (*b* = -0.20, *SE* = 0.06, *t*(122) = -3.51, *p* < .001). This interaction indicates that the correlation was strongest for men looking at female faces (*r* = .32), whereas weaker correlations were found for women looking at female faces (*r* = .14) and women looking at male faces (*r* = .11). The weakest correlation was found for men looking at male faces (*r* = .05). Finally, we found a Relationship Status × Sociosexual Orientation interaction (*b* = 0.05, *SE* = 0.02, *t*(124) = 2.15, *p* = .034). The interaction shows a dissociation regarding the role of sociosexual orientation between participants in a relationship and single participants. For participants in a relationship, an increase towards a more unrestricted sociosexual orientation was related to a decrease in the correlation; in other words, the more unrestricted their sociosexual orientation, the smaller the effect of attractiveness on TFD. For single participants on the other hand, an increase towards a more unrestricted sociosexual orientation was related to an increase in the correlation. For these participants, the more unrestricted their sociosexual orientation was, the higher was the effect of attractiveness on TFD (see Fig 2).

### Mixed-sex scenes

For mixed-sex scenes, all correlation coefficients were positive (see Table 1). Reflecting the same pattern as in same-sex scenes, we found a Participant Sex × Sex of Face interaction (*b* = -0.17, *SE* = 0.07, *t*(140) = -2.24, *p* = .027). The strongest correlation was found for men looking at female faces (*r* = .23). Weaker correlations were found for women looking at female faces (*r* = .15) and women looking at male faces (*r* = .17). The weakest correlation was again found for men looking at male faces (*r* = .09).

## Discussion

We showed how relationship status and sociosexual orientation moderate the link between facial attractiveness and visual attention. The eye-tracking results replicated the previously found positive correlation between attractiveness and total fixation duration (TFD) [10–14, 16–18]. Across all conditions, we found main effects of attractiveness, which replicates findings showing that attractive faces are looked at longer [10, 12]. This is in accordance with the idea that looking at attractive faces is rewarding and elicits positive emotions [72–76]. Put simply, we tend to look at attractive faces because we enjoy looking at them.

Most interesting regarding our research question is the interaction between relationship status and sociosexual orientation (same-sex scenes; see Fig 2). For single participants, we found that an increase towards a more unrestricted sociosexual orientation is associated with an increase in TFD in combination with an increase in attractiveness. This confirms our hypothesis that attractiveness is more important to people who are more sociosexually unrestricted [61]. Moreover, the positive correlation between attractiveness and TFD was lower for participants in a relationship and who were more sociosexually unrestricted compared to those who were less unrestricted. According to the idea of relationship maintenance, we hypothesized that irrespective of sociosexual orientation, participants in a relationship would show a low and stable positive correlation. However, the opposite was the case in that the correlation decreased with an increase in sociosexual unrestrictedness. A possible explanation of why the mean correlations were not the same for more and less sociosexually unrestricted participants in a relationship is that being in a relationship has different implications for both groups. Being highly sociosexually unrestricted implies that a person is interested in engaging in a number of short-term relationships. However, being in a relationship at the same time could lead to strong self-restrictions to avoid potential temptations and endangering the relationship.

For single participants who were also more sociosexually unrestricted, the increase in TFD was clearly steeper than the decrease in TFD for participants in a relationship. Therefore, it seems that for single participants, sociosexual orientation is more influential than for participants in a relationship. Fig 2 further shows that the most sociosexually unrestricted single participants showed higher correlations than any other group. This result is in line with the finding that attractiveness is especially important for short-term relationships [57–59]. Furthermore, for single participants, favoring attractiveness is comparatively more important and beneficial as they are more likely looking for a partner. Fig 2 and Table 1 show that the least sociosexually unrestricted single participants showed the smallest correlations. We expected that single participants, in general, would have at least moderate correlations and that the correlations would become stronger with an increase in unrestricted sociosexual orientation. A possible explanation of why less sociosexually unrestricted single participants do not show this pattern is that some of them were not looking for a partner at the time they participated in the study. Thus, attractiveness did not increase their attention to the same extent as for those who were looking for partners. Consequently, the attractiveness-attention correlations were smaller. In addition, only a few of the face stimuli were of high attractiveness. Therefore, it is likely that only a few faces increased attention to a great extent.

Inspection of Table 1 shows no clear evidence for relationship maintenance processes. Comparing the correlations of single participants with those in a relationship—when considering both types of scenes—shows that the mean correlations were higher for men in a relationship than single men, whereas single women showed a slightly higher correlation than women in a relationship (see Table 1). We expected these results for women [14], although we would have expected bigger differences between the groups. The results for men can be interpreted in terms of the evolutionary notion that a person will continue looking for better alternatives even if she/he is already in a relationship. There is evidence that single participants as well as those in a relationship—to whom their relationship status is not made salient—look at attractive faces of the other sex [38, 39, 41, 42, 77]. Other factors such as the length of time that someone is in a relationship, how secure one feels about the relationship, attractiveness of the partner, and relationship satisfaction are just some factors that could explain these mixed results. Our result that an increase in sociosexual unrestrictedness for people in a relationship led to a decrease in the correlation suggests that sociosexual orientation could moderate relationship maintenance behavior. This and the effects of the other mentioned factors would need to be examined in future research.

Regarding the sex of the faces and the sex of the participants, we found that the main effect of attractiveness on TFD was moderated by the sex of the face stimulus (same-sex scenes). In same-sex scenes, the perceiver can directly compare the attractiveness of the two same-sex faces. Accordingly, same-sex scenes are more informative in terms of mate selection. Moreover, this correlation was also generally stronger for female faces than for male faces. We also found an interaction between the sex of the perceiver and the sex of the face stimulus (for same-sex and mixed-sex scenes). The correlation was strongest for men looking at female faces. These findings are in line with studies that have shown that men place greater emphasis on physical attractiveness of the other sex than women do [53–56, 64, 65]. However, we did not find support for the “*opposite-sexed beauty captures the mind*” hypothesis as our data tend to support the “*one-sided gender bias*” hypothesis [14] and the well-established finding that women distribute their attention quite evenly between the two sexes, whereas (heterosexual) men clearly prefer looking at female faces [78, 79]. Actually, both men and women looked longer at female faces. In addition, attractive female faces were looked at longer, no matter whether a woman is presented next to another woman or next to a man. This is support for the “*female beauty captures the mind*” hypothesis [14].

Previous studies have suggested that making the relationship status salient to the participants changes their behavior. Consequently, it would be interesting to see whether the results involving eye movements—which we consider as a fairly objective measure—would differ if participants’ relationship status were brought to their attention. Research regarding relationship maintenance has already shown that early cognitive processes are influenced by relationship status. Thus, making relationship status salient could lead to even bigger differences between single participants and those who are in a relationship. Additionally, future studies could make participants’ relationship status salient by having them perform a specific task, such as having them choose a potential partner in a forced-choice paradigm. Including such a task could more strongly differentiate the attractiveness-attention correlation between single participants and those in a relationship.

Other variables that we did not include in this study, but which could be of interest for future studies are participants’ desire to have children, how secure they feel about their relationship, how stable they perceive their relationship to be, how satisfied they are with their relationship, how committed they are to their relationship, how strong/weak their attachments to their partners are, and whether they are motivated to seek out and meet people of the other sex. A further limitation of the study is that we assessed relationship status as a dichotomous variable: single or being in a romantic relationship. It could have been more informative to include options such as exclusively dating, single but dating, single but not dating, living together, dating steadily, and dating occasionally [29].

To summarize, the present study has shown that the attractiveness-attention correlation is stronger for other-sex faces than for same-sex faces. In same-sex scenes, we found clear support for the hypothesis that female beauty increases visual attention. However, the attractiveness-attention correlation is moderated by relationship status and sociosexual orientation. This shows that perceiver characteristics, goals, and motives moderate the adaptive function of attractive faces on attention. Thus, evolutionary notions alone cannot explain the link between attractiveness and attention.

## Supporting information

S1 Appendix Results–larger LMMs. Reasoning for and results of additional LMMs using total fixation duration as dependent variable and including attractiveness as an independent factor.
(DOCX)
