# Supplementary material for: How relationship status and sociosexual orientation influence the link between facial attractiveness and visual attention
Source: PLoS One. 2018 Nov 14;13(11):e0207477. doi: 10.1371/journal.pone.0207477 (PMC6241135; doi:10.1371/journal.pone.0207477)
Supplement: S1 Appendix — Reasoning for and results of additional LMMs using total fixation duration as dependent variable and including attractiveness as an independent factor. (DOCX) [file pone.0207477.s001.docx]

**S1 Appendix. Results – larger LMMs.**

In comparison to the “smaller” LMMs, where we used the correlation between attractiveness ratings and total fixation duration as dependent variable, in the “larger” LMMs reported here we used the total fixation duration (TFD) as dependent variable. The integration of attractiveness and TFD into one variable has the advantage of reducing the number of factors by one, making the statistical models more concise and easier to interpret. However, in doing so, variation within participants is lost, potentially reducing the reliability and power of the analyses. Therefore, we ran two additional LMMs (one per scene type, same-sex scenes see deposited data “o:756909”; mixed-sex scenes see deposited data “o:756910”) using TFD as dependent variable with attractiveness as an independent factor (centered, continuous) and contrasts for the fixed effects of the sex of the participant (women – men), sex of face stimulus (female – male), and relationship status (single – committed). We included sociosexual orientation as a centered, continuous fixed effect, and interactions among all factors. We also included a random by-face intercept and slopes for participant sex, attractiveness, relationship status, and sociosexual orientation and a by-participant intercept and slopes for attractiveness and sex of face. It is important to note that in these additional, larger LMMs, only those effects are relevant for our research questions that included attractiveness. Consequently, we report only these.

**Same-sex Scenes**

We found a main effect of attractiveness with TFD as the dependent variable (*b* = 132.42, *SE* = 16.73, *t*(128) = 7.92, *p* < .001). Attractive faces were looked at longer compared to less attractive faces. We found an Attractiveness × Participant Sex interaction (*b* = -83.68, *SE* = 29.87, *t*(147) = -2.80, *p* = .006). For men, an increase in attractiveness led to a steep increase in TFD, so the difference between least and most attractive faces was pronounced, whereas for women the difference was much smaller. We found an Attractiveness × Sex of Face interaction (*b* = 83.04, *SE* = 24.63, *t*(82) = 3.37, *p* = .001). The increase in TDF from low to high attractive faces was much smaller for male faces compared to female faces. We found an Attractiveness × Participant Sex × Sex of Face interaction (*b* = -126.71, *SE* = 41.39, *t*(772) = -3.06, *p* = .002). This interaction indicates that the increase in TFD with an increase in attractiveness was largest for men looking at female faces. We found an Attractiveness × Relationship Status × Sociosexual Orientation interaction (*b* = 51.40, *SE* = 19.22, *t*(135) = 2.67, *p* = .008). For single participants, an increase in sociosexual unrestrictedness led to an increase in TFD, except for the least attractive faces for which an increase in unrestrictedness led to a decrease in TFD. For participants in a relationship, the pattern is reversed: for attractive faces, an increase in unrestrictedness led to a decrease in TFD, whereas for less attractive faces, the increase in unrestrictedness led to an increase in TFD. No other effects including attractiveness reached significance.

**Mixed-sex Scenes**

We found a main effect of attractiveness (*b* = 164.95, *SE* = 24.89, *t*(76) = 6.23, *p* < .001). Attractive faces were looked at longer than less attractive faces. We found an Attractiveness × Participant Sex × Sex of Face interaction (*b* = -135.17, *SE* = 60.61, *t*(1376) = -2.23, *p* = .026), which followed the pattern in same-sex scenes, namely, the increase in attractiveness and relatedly TFD was strongest for men looking at female faces. For all other combinations of sex, we also found an increase in TFD with an increase in attractiveness. However, it was less pronounced compared to men looking at female faces. No other effects including attractiveness reached significance.

Table S1

*Results for the Larger LMM with TFD as Dependent Variable Separately for Same-sex and Mixed-sex Scenes*

|  | Same-sex scenes | | | | | Mixed-sex scenes | | | | |
| --- | --- | --- | --- | --- | --- | --- | --- | --- | --- | --- |
|  | Est. | *SE* | *df* | *t* | *p* | Est. | *SE* | *df* | *t* | *p* |
| Intercept | 2903 | 58 | 179 | 50.31 | <.001* | 2928 | 69 | 84 | 42.23 | <.001* |
| Attractiveness | 132 | 17 | 128 | 7.92 | <.001* | 165 | 25 | 76 | 6.63 | <.001* |
| Sex of the participant | 294 | 96 | 149 | 3.06 | .003* | 327 | 102 | 141 | 3.21 | .002* |
| Sex of the face | 61 | 74 | 67 | 0.82 | .414 | 438 | 109 | 38 | 4.02 | <.001* |
| Relationship status | -184 | 95 | 143 | -1.94 | .054† | -135 | 103 | 137 | -1.31 | .194 |
| Sociosexual orientation (SOI) | 14 | 32 | 145 | 0.45 | .653 | 15 | 34 | 140 | 0.44 | .659 |
| Attractiveness : Sex of the participant | -84 | 30 | 147 | -2.80 | .006* | -59 | 40 | 141 | -1.49 | .137 |
| Attractiveness : Sex of the face | 83 | 25 | 82 | 3.37 | .001* | 64 | 42 | 49 | 1.54 | .129 |
| Sex of the participant : Sex of the face | -46 | 75 | 73 | -0.62 | .538 | 89 | 110 | 122 | 0.82 | .417 |
| Attractiveness : Relationship status | -26 | 29 | 133 | -0.90 | .370 | -17 | 40 | 132 | -0.42 | .677 |
| Sex of the participant : Relationship status | 358 | 189 | 142 | 1.90 | .060† | 344 | 203 | 141 | 1.70 | .092† |
| Sex of the face : Relationship status | 18 | 66 | 90 | 0.27 | .790 | -141 | 115 | 77 | -1.23 | .223 |
| Attractiveness : SOI | 10 | 10 | 136 | 1.06 | .291 | 12 | 13 | 155 | 0.92 | .361 |
| Sex of the participant : SOI | 72 | 63 | 143 | 1.14 | .257 | 56 | 68 | 140 | 0.83 | .410 |
| Sex of the face : SOI | 10 | 22 | 124 | 0.44 | .664 | -21 | 37 | 118 | -0.57 | .569 |
| Relationship status : SOI | 39 | 63 | 143 | 0.63 | .532 | 62 | 68 | 140 | 0.92 | .361 |
| Attractiveness : Sex of the participant : Sex of the face | -127 | 41 | 772 | -3.06 | .002* | -135 | 61 | 1376 | -2.23 | .026* |
| Attractiveness : Sex of the participant : Relationship status | 73 | 58 | 138 | 1.27 | .205 | -75 | 79 | 143 | -0.95 | .344 |
| Attractiveness : Sex of the face : Relationship status | 10 | 39 | 874 | 0.24 | .807 | -90 | 62 | 514 | -1.47 | .142 |
| Sex of the participant : Sex of the face : Relationship status | -262 | 128 | 137 | -2.05 | .042* | 42 | 217 | 136 | 0.20 | .846 |
| Attractiveness : Sex of the participant : SOI | 10 | 19 | 135 | 0.50 | .615 | 32 | 27 | 155 | 1.18 | .241 |
| Attractiveness : Sex of the face : SOI | 24 | 13 | 2740 | 1.86 | .063† | -20 | 21 | 1855 | -0.94 | .346 |
| Sex of the participant: Sex of the face : SOI | -16 | 43 | 134 | -0.38 | .706 | -86 | 73 | 135 | -1.18 | .241 |
| Attractiveness : Relationship status : SOI | 51 | 19 | 135 | 2.67 | .008* | -35 | 27 | 154 | -1.32 | .190 |
| Sex of the participant : Relationship status : SOI | -118 | 126 | 143 | -0.94 | .348 | -124 | 136 | 140 | -0.91 | .363 |
| Sex of the face : Relationship status : SOI | -61 | 43 | 134 | -1.44 | .153 | 171 | 73 | 134 | 2.35 | .020* |
| Attractiveness : Sex of the participant : Sex of the face : Relationship status | 32 | 78 | 3207 | 0.41 | .686 | -37 | 120 | 2561 | -0.31 | .757 |
| Attractiveness : Sex of the participant : Sex of the face : SOI | -38 | 26 | 3245 | -1.45 | .147 | -11 | 41 | 2621 | -0.28 | .782 |
| Attractiveness : Sex of the participant : Relationship status : SOI | -35 | 38 | 135 | -0.92 | .361 | -3 | 54 | 154 | -0.06 | .953 |
| Attractiveness : Sex of the Face : Relationship status : SOI | 15 | 26 | 3261 | 0.60 | .552 | -20 | 41 | 2624 | -0.50 | .620 |
| Sex of the participant : Sex of the face : Relationship status : SOI | 149 | 85 | 134 | 1.76 | .082† | -56 | 146 | 134 | -0.38 | .702 |
| Attractiveness : Sex of the participant : Sex of the face : Relationship status : SOI | 39 | 52 | 3263 | 0.76 | .447 | -97 | 83 | 2624 | -1.18 | .239 |

*Note.* * p < 0.05, † p < 0.10. SOI: Sociosexual orientation. Contrasts: sex of the participant (women – men); sex of the face (female faces – male faces); relationship status (single – committed).
